# Genoprotective activities of plant natural substances in cancer and chemopreventive strategies in the context of 3P medicine

**DOI:** 10.1007/s13167-020-00210-5

**Published:** 2020-05-29

**Authors:** Lenka Koklesova, Alena Liskova, Marek Samec, Tawar Qaradakhi, Anthony Zulli, Karel Smejkal, Karol Kajo, Jana Jakubikova, Payam Behzadi, Martin Pec, Pavol Zubor, Kamil Biringer, Taeg Kyu Kwon, Dietrich Büsselberg, Gustavo R. Sarria, Frank A. Giordano, Olga Golubnitschaja, Peter Kubatka

**Affiliations:** 1 Department of Obstetrics and Gynecology, Jessenius Faculty of Medicine, Comenius University in Bratislava, 036 01 Martin, Slovakia; 2 Institute for Health and Sport, Victoria University, Melbourne, VIC Australia; 3 Department of Natural Drugs, Faculty of Pharmacy, Masaryk University, 612 42 Brno, Czech Republic; 4 Department of Pathology, St. Elisabeth Oncology Institute, 812 50 Bratislava, Slovakia; 5 Biomedical Research Center, Slovak Academy of Sciences, 845 05 Bratislava, Slovakia; 6 Biomedical Research Center SAS, Cancer Research Institute, Bratislava, Slovakia; 7 Department of Microbiology, College of Basic Sciences, Shahr-e-Qods Branch, Islamic Azad University, Tehran, Iran; 8 Department of Medical Biology, Jessenius Faculty of Medicine, Comenius University in Bratislava, 03601 Martin, Slovakia; 9 Department of Gynecologic Oncology, Norwegian Radium Hospital, Oslo University Hospital, Oslo, Norway; 10 OBGY Health & Care, Ltd., 01001 Zilina, Slovakia; 11 Department of Immunology and School of Medicine, Keimyung University, Dalseo-Gu, Daegu, 42601 Korea; 12 Department of Physiology and Biophysics, Weill Cornell Medicine-Qatar, Education City, Qatar Foundation, P.O. Box 24144, Doha, Qatar; 13 Department of Radiation Oncology, University Hospital Bonn, Rheinische Friedrich-Wilhelms-Universität Bonn, Bonn, Germany; 14 Predictive, Preventive and Personalised (3P) Medicine, Department of Radiation Oncology, University Hospital Bonn, Rheinische Friedrich-Wilhelms-Universität Bonn, Bonn, Germany

**Keywords:** Predictive Preventive Personalised Medicine (3PM, PPPM), Oncology, Tumour, Plant natural substances, Phytochemicals, Antimutagenic effects, Genomic instability, Genotoxicity, Prebiotic, Probiotic, Anti-inflammatory, Antioxidant, Antibacterial, Antifungal, Anticancer, Beneficiary effects, Genoprotection, Chemoprevention, Superoxide dismutase, Hydrogen peroxide, Glutathione, Thioredoxin, Glutaredoxins, ROS, Scavanger, Preclinical and clinical study, Exogenous and endogenous agents, Oxidative stress, Biomarkers, Therapeutic potential, Diet, Nanoparticles, Nanotechnology, Detoxification, Breast cancer, Colon cancer

## Abstract

Severe durable changes may occur to the DNA structure caused by exogenous and endogenous risk factors initiating the process of carcinogenesis. By evidence, a large portion of malignancies have been demonstrated as being preventable. Moreover, the targeted prevention of cancer onset is possible, due to unique properties of plant bioactive compounds. Although genoprotective effects of phytochemicals have been well documented, there is an evident lack of articles which would systematically present the spectrum of anticancer effects by phytochemicals, plant extracts, and plant-derived diet applicable to stratified patient groups at the level of targeted primary (cancer development) and secondary (cancer progression and metastatic disease) prevention. Consequently, clinical implementation of knowledge accumulated in the area is still highly restricted. To stimulate coherent co-development of the dedicated plant bioactive compound investigation on one hand and comprehensive cancer preventive strategies on the other hand, the current paper highlights and deeply analyses relevant evidence available in the area. Key molecular mechanisms are presented to detail genoprotective and anticancer activities of plants and phytochemicals. Clinical implementation is discussed. Based on the presented evidence, advanced chemopreventive strategies in the context of 3P medicine are considered.

## Introduction

The traditional use of medicinal plants led to the discovery of numerous bioactive compounds representing potent tools in the therapy approaches and disease prevention [1]. Major benefits of isolated chemical compounds or their mixtures found in fruits, vegetables, beans, and other plant sources include anti-inflammatory, antioxidant, antibacterial, antifungal, and other health beneficiary effects [2–6].

Recently, plant natural substances are at the center of scientific interest due to their anticancer activity as described elsewhere [2, 7, 8]. For instance, an evidence suggests a correlation between higher consumption of phytochemical-rich foods and lower risk of cancer development [9–11]. Additionally, the antineoplastic efficacy of phytochemicals can be mediated via the maintenance of genome stability. Genotoxicity can be interpreted as harmful genetic changes such as gene mutations, recombination, or chromosomal aberrations in the presence of genotoxins action [12]. Multiple genomic events as a consequence of carcinogen exposures or disbalance of anti and prooxidative reactions characterize tumour evolution [13]. Healthy cells have evolved mechanisms to inactivate free radicals or carcinogenic chemicals and thus prevent DNA damage [14]. On the other hand, the imbalance and/or deficits within DNA-repair cascades can be associated with initiation, promotion, and progression of carcinogenesis [15]. Therefore, the human environment and dietary habits play a crucial role in cancer etiology [16].

Despite the use of various anticancer drugs which improve symptoms and increase patient survival, many cancers remain incurable, mainly due to advanced cancer stages diagnosed, deficient patient stratification, and neglected individualised patient profiles [17]. As such, introducing new clinical approaches reducing cancer risks would be highly beneficial for healthcare as a whole. To this end, instability of DNA structure as critical aspect of carcinogenesis under certain conditions can be chemoprevented by plant-derived bioactive compounds (phytochemicals) resulting in reduced risks of cancer development [18–26]. This review highlights central mechanisms of phytochemicals as natural genoprotectors, presents a spectrum of their anticancer properties, and considers their potential application for individuals at risk and patients in the context of 3P medicine, namely, predictive diagnostics, targeted primary and secondary prevention, and personalisation of treatments.

## Sources of data used

English-language biomedical literature sources from PubMed database were analysed for the topic-related items including all the keywords listed above. The most recent scientific publications originated from years 2015–2020 were particularly taken into consideration for the final statement presented in the paper.

## DNA damaging agents

**Fig. 1:**
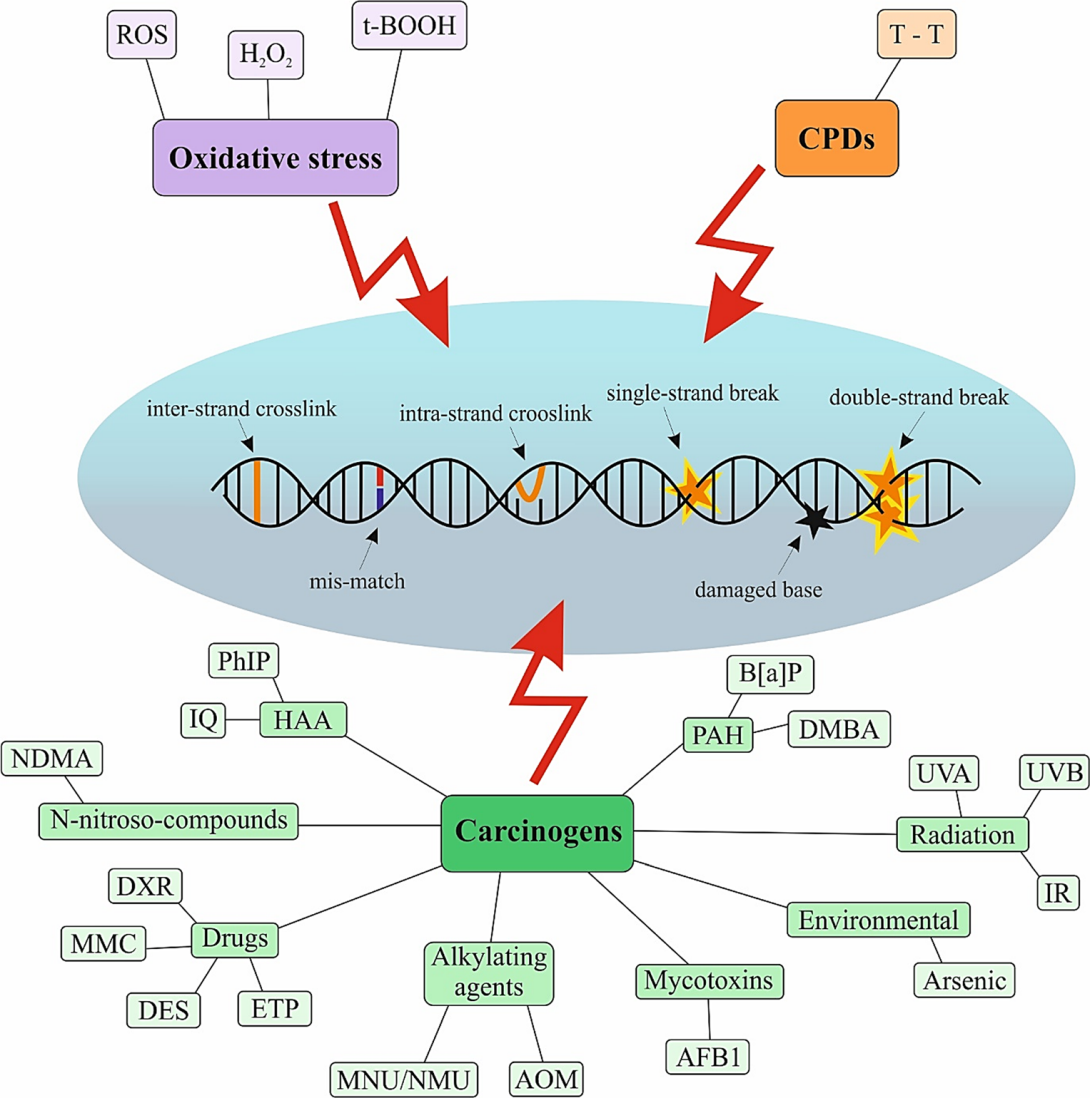
Endogenous and exogenous agents causing DNA damage. Oxidative stress, production of cyclobutane pyrimidine dimers, or carcinogens exposure act as the main initiators of DNA damage. ROS, reactive oxygen species; H_2_O_2_, hydrogen peroxide; CPDs, cyclobutane pyrimidine dimers; T-T, thymine dimers; HAA, heterocyclic aromatic amines; PAH, polycyclic aromatic hydrocarbons; PhIP, 2-amino-1-methyl-6-phenylimidazo[4,5-b]pyridine; IQ, 2-amino-3-methylimidazo[4,5-f]quinoline; NDMA, N-nitrosodimethylamine; DXR, doxorubicin; MMC, mitomycin C; DES, diethylstilbestrol; ETP, etoposide; MNU/NMU, N-methyl-N-nitrosourea; AOM, azoxymethane; AFB1, aflatoxin B1; IR, ionising radiation; UVA, ultraviolet A; UVB, ultraviolet B; DMBA, 7,12-dimethylbenz[a]anthracene; B[a]P, benzo[a]pyrene

Carcinogenesis is associated with extensive DNA damage, which is often caused by an exposure to various exogenous and endogenous agents. Thus, maintaining genomic integrity is crucial for the well-being of the organism [27]. Medication applying DNA damaging agents against cancer utilizes the biologic difference in the response between normal and tumour cells towards DNA injury, due to highly increased proliferation of the latter [28]. Figure 1 highlights exogenous and endogenous agents causing DNA damage.

### Oxidative stress and reactive oxygen species (ROS)

Oxidative stress is caused by an imbalance between ROS formation and scavenging. Increased production of ROS has been detected in various cancer types with important roles in the activation of pro-tumourigenic signalling, enhancement of cell survival, and proliferation or drive of DNA damage and genetic instability [29]. Modifications of nitrogen bases in DNA or its sugar-phosphate backbone can be caused by ROS, which disrupt gene function and impair transcription, DNA replication, and cell proliferation [30]. Hydrogen peroxide (H_2_O_2_) is a relatively weakly reactive ROS and acts as a mild oxidising/reducing agent. H_2_O_2_ can oxidize DNA, lipids, and proteins mainly in increased presence of hyperreactive thiol groups or methionine residues [31]. In addition, DNA damage can be caused by inducers of ROS such as tert-butyl hydroperoxide (t-BOOH) [32]. The level of DNA damage arising from oxidative stress can be measured by analysis of level of 8-hydroxy-2′-deoxyguanosine (8-OHdG) or 8-oxo-7,8-dihydro-2′-deoxyguanosine (8-oxodG), which are widely used biomarkers for oxidative stress and carcinogenesis [33].

### Cyclobutane pyrimidine dimers

An accumulation of UV-specific mutations caused by direct absorption of UV photons, such as pyrimidine dimers, is closely associated with melanoma skin cancer. UVA-induced cyclobutane pyrimidine dimers (CPDs) are formed between adjacent thymine bases, by either direct excitation or photosensitisation leading to disruption of the normal cellular processing of DNA. This DNA damage causes various biological responses including apoptosis, immune suppression, and carcinogenesis [34, 35].

### Exposure to carcinogens

Exposure to carcinogens is associated with occurrence of electrophiles or ROS and can result in the cancer initiation and promotion. Carcinogens cause damage of DNA including single-strand breaks (SSBs) or double-strand breaks (DSBs), oxidative-induced lesions, covalently bound DNA chemical adducts, and DNA-DNA or DNA-protein cross-links [36]. Carcinogens are represented by heterocyclic aromatic amines (tobacco smoke, diesel exhaust, incineration ash), polycyclic aromatic hydrocarbons (roasting, grilling, baking, smoking), ultraviolet (UV) and infrared (IR) radiation, aristocholic acid, nitrosamines (drugs, cosmetics, rubber industry), mycotoxins, asbestos, and some nanoparticles [36, 37]. Specific examples of carcinogens and their DNA damaging mechanisms are summarised in Table 1.

**Table 1:** Specific groups of carcinogens and their DNA damaging mechanisms

Groups	Carcinogens	Mechanisms of DNA damage	References
Radiation	UVA	Direct formation of DNA lesions, oxidation, and damage to DNA repair proteins	[38]
	UVB	↑ CPDs	[39]
	IR	↑ DSBs, secondary effects: generation of abasic sites and SSBs	[40]
PAH	B[a]P	↑ B[a]P diol epoxidation (BPDE) and BPDE–DNA adducts	[41]
	DMBA	↑ Covalent adducts with DNA, formation of depurinated abasic sites within DNA	[42]
HAA	PhIP	↑ DNA adducts: PhIP-C8-dG	[37]
	IQ	↑ IQ-DNA adducts	[43, 44]
N-nitroso-compounds	NDMA	↑ DNA adducts, the conversion into methylamines and induction of o-methylguanine	[45]
Drugs	DXR	↑ DNA DSBs and DNA adducts, ↑ oxygen free radicals	[46]
	MMC	Alkylation DNA, generation DNA cross-links	[47]
	DES	↑ DES-DNA adducts (3′-OH-DES-6′-N3Ade and 3′-OH-DES-6′-N7Gua)	[48]
	ETP	Topoisomerase II inhibitor, ↑ DNA DSBs	[49]
Alkylating agents	MNU/NMU	↑ DNA methylation adducts O(6)-methylguanine, minor products: O2-methylcytosine (O2MeC) and O2-methylthymine (O2MeT)	[50, 51]
	AOM	Mutation in exon 3of *Ctnnb* gene, constitutive activation of the Wnt pathway	[52]
Mycotoxins	AFB1	↑ AFB1-DNA adducts (AFB1-N(7)-guanine)	[53, 54]
Environmental	Arsenic	DNA repair inhibition, gene expression alterations via epigenetic modifications	[55]

*PAH* polycyclic aromatic hydrocarbon; *HAA* heterocyclic aromatic amines; *UVA* ultraviolet A; *UVB* ultraviolet B; *IR* ionising radiation; *B[a]P* benzo[a]pyrene; *DMBA* 7,12-dimethylbenz[a]anthracene; *PhIP* 2-amino-1-methyl-6-phenylimidazo[4,5-b]pyridine; *IQ* 2-amino-3-methylimidazo[4,5-f]quinoline; *NDMA* N-nitrosodimethylamine; *DXR* doxorubicin; *MMC* mitomycin C; *DES* diethylstilbestrol; *ETP* etoposide; *MNU/NMU* N-methyl-N-nitrosourea; *AOM* azoxymethane; *AFB1* aflatoxin B1; *CPDs* cyclobutane pyrimidine dimers; *DSBs* double-strand breaks; *SSBs* single-strand breaks

↑increase/induce

## Molecular mechanisms involved in genoprotective action

Damage of DNA is essential for the induction of mutations associated with initiation, promotion, and progression of carcinogenesis [15]. Accordingly, healthy cells must be defended against DNA damage caused by endogenous and exogenous agents and decrease the mutagenic processes. As discussed below, molecular mechanisms of genoprotective agents are involved in the protection against DNA damage.

### Antioxidant activity

ROS directly oxidize DNA and interfere with various mechanisms of DNA repair, triggering DNA chain breaks, base modification, and other oxidative DNA lesions [56]. In cancer management, ROS scavenging is an important antioxidant mechanism with an effort to reduce tumour growth and survival of cancer cells and can be achieved by different types of plant-derived phytochemicals [57–59]. ROS can be scavenged via either enzymatic or non-enzymatic pathways such as glutathione (GSH), thioredoxin (Trx), superoxide dismutase (SOD), catalase (CAT), and peroxidases [60, 61].

Cellular defense against oxidative stress is mediated mainly by an activation of the Nrf2-antioxidant response element signalling pathway. Under physiological conditions, the Nrf2 expression is regulated through the binding to Keap1 which is associated with Nrf2 degradation [62]. On the contrary, upon oxidative stress, this binding is interrupted and Nrf2 can translocate to the nucleus and bind an antioxidant response element (ARE) sequences that are present in enhancer regions of cytoprotective genes. Subsequently, these genes encode enzymes and proteins with the function in the balance of redox homeostasis and detoxification oxidants or electrophiles. Furthermore, cytoprotective genes code an ability to remove or repair damaged DNA and proteins [63–65].

Nrf2-independent pathway is involved in the protection against ROS-induced DNA damage. Aryl hydrocarbon receptor (AhR) is defined as ligand-activated transcription factor, which binds to exogenous ligands leading to nuclear translocation of AhR and consequent dimerisation with associated AhR protein. A subsequent interaction between the heterodimer with consensus DNA sequence xenobiotic responsive element and the enhancer regions of target genes increases their transcription [37, 66]. Inactivation of carcinogens, such as B[a]P by phytochemicals, can lead to the protection against DNA damage as was demonstrated in several studies concerning cancer chemoprevention and therapy [67–69].

Under specific cellular conditions, antioxidants can also act as prooxidants, which promote production of ROS, subsequently cause different DNA damage and initiate mutagenesis. To determine the prooxidant status, the concentration of several reductant-oxidant markers, including glutathione (GSH) to GSSG, NADPH to NAPD^−^, and NADH to NAD^−^, has to be evaluated [70]. The prooxidant activity is catalysed by metals, especially transition metals present in biological systems such as iron (Fe) and copper (Cu) [71]. The prooxidant effect was observed in numerous phenolic compounds including flavonoids [72, 73]. Moreover, cytotoxic level of ROS caused by prooxidant compounds is increased in cancer cells probably due to the higher concentration of iron/copper ions and greater metabolic activity compared with normal cells [74]. Inhibition of prooxidative enzyme activation, including the GSH, thioredoxin (TXN), or NADPH oxidase, can represent a potential target in cancer prevention and treatment [75, 76].

### Detoxification processes

In mammals, metabolic activation of phase I and phase II enzymes is involved in liver detoxification of various classes of environmental carcinogens. These enzymes can enhance the elimination of carcinogens and protect DNA against damage [77, 78]. Cytochrome P450 (CYP) is the main enzyme of phase I detoxification, which converts xenobiotics to active intermediates [79]. In tumour tissues, CYP cause resistance by metabolising and, therefore, targetly deactivating the cytostatics [80]. Phase II detoxification enzymes, including glutathione S-transferases (GSTs), NAD(P)H:quinone oxidoreductase 1 (NQO1), and heme oxygenase-1 (HO-1), catalyze the conjugation of these active intermediates by sulfation or glucuronidation [81]. Moreover, GST activity can apparently influence DNA stability and repair process towards oxidised bases [82].

**Fig. 2:**
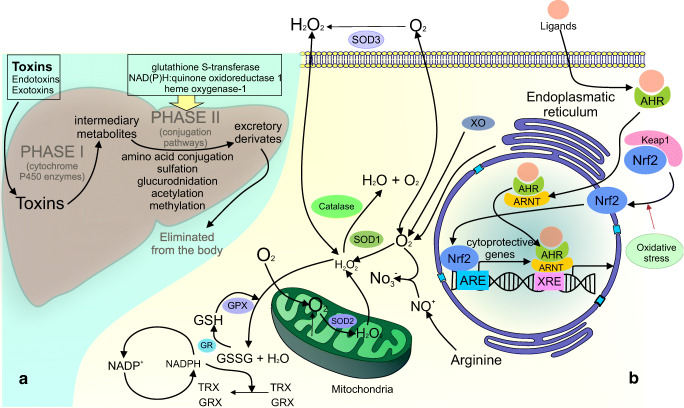
Antioxidant and detoxifying processes involved in genoprotective activities. Part A demonstrates the metabolic processes of phase I (Cytochrome P450) and phase II (GSTs, NQO1, HO-1) enzymes involved in detoxification of environmental carcinogens. Part B describes the ROS scavenging activity via either enzymatic or non-enzymatic pathways, Nrf2-antioxidant response element signalling pathway, and AhR-dependent pathway. SOD1, superoxide dismutase 1; SOD2, superoxide dismutase 2; SOD3, superoxide dismutase 3; H2O2, hydrogen peroxide; GSH, glutathione; GR, glutathione reductase; GSSG, glutathione disulfide; NADP, nicotinamide adenine dinucleotide phosphate; XO, xanthine oxidase; AhR, aryl hydrocarbon receptor; Nrf2, nuclear factor erythroid 2-related factor 2; KEAP1, Kelch-like ECH-associated protein 1; TRX, thioredoxin; GRX, glutaredoxins; ARE, antioxidant response element; XRE, xenobiotic response element

Cellular mechanisms of antioxidant defense system and detoxification are included in Fig. 2.

### DNA repair mechanisms

In case of a mild damage of DNA (the repair of DNA is possible), various signalling cascades are activated by cells to restore the original genetic information. Genetic mutations in genes coding the DNA repair may contribute to the cancer initiation. DNA damages, such as DSBs and SSBs, activate pair of related protein kinases ATM and ATR, respectively. In case of mutations, ATM and ATR phosphorylate several common substrates leading to an initiation of a cascade resulting in cell cycle arrest and DNA repair [83].

The simplest process to repair SSBs induced by ROS is base excision repair (BER). DNA repair of base damage via BER is provided by four enzymes including a DNA glycosylase, AP-endonuclease I (APE1), DNA polymerase β (Polβ), and a DNA ligase [84, 85]. OGG1 is a repair enzyme 8-oxoGua DNA glycosylase 1 that removes the oxidised purine from DNA as the first step in BER. AP endonuclease bypasses the AP lyase activity of OGG1, thus enhancing OGG1 turnover (APE1) [18]. Moreover, other repair mechanisms, such as two types of nucleotide excision repair (NER) and DNA mismatch repair (MMR), are closely linked to cancer cells. In transcription-coupled nucleotide excision repair (TC-NER), DNA damage is recognised by XPC enzyme and the double strand of DNA is subsequently unfolding from both sides through the presence of helicases XPA, XPG, and TFIIH. Damaged DNA is removed and the synthesis of a new strand can start [86]. Furthermore, other global genomic nucleotide excision repair (GG-NER) mechanisms may repair damage in transcribed or untranscribed DNA strands throughout the genome [87]. Small loops in DNA can be recognised and repaired by MMR either by base-base mismatches or by insertion/deletion loops [88]. NER and BER systems repair damages affecting just SSBs and the lesions involving exogenous and endogenous sources, respectively [89].

**Fig. 3:**
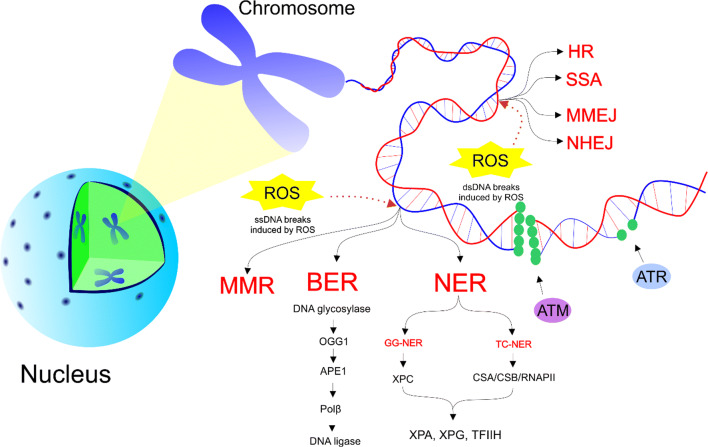
Repair mechanisms involved in genoprotective activities. Mutation causes the phosporylation of substrates by ATM and ATR recognising DSBs and SSBs, respectively. SSBs are associated with three different repair mechanisms including NER (TC-NER and GG-NER), BER, and MMR. DSBs can be repaired primarily via error-free HR but in case of defect of HR, error-prone SSA, NHEJ, or MMEJ are involved in repair. ssDNA, single-strand DNA; dsDNA, double-strand DNA; ROS, reactive oxygen species; HR, homologous recombination; SSA, single-strand annealing; MMEJ, microhomology-mediated end joining; NHEJ, non-homologous end joining; MMR, DNA mismatch repair; BER, base excision repair; NER, nucleotide excision repair; ATM, protein kinase ataxia-telangiectasia mutated; ATR, ataxia telangiectasia and Rad3-related protein; OGG1, 8-oxoguanine glycosylase; APE1, human apurinic/apyrimidinic endonuclease 1 Polβ, polymerase β; GG-NER, global genomic nucleotide excision repair; TC-NER, transcription-coupled nucleotide excision repair; XPC, CSA, Cockayne syndrome group A protein; CSB, Cockayne syndrome group B protein; RNAPII, RNA polymerase II; XPA, DNA repair protein complementing xeroderma pigmentosum-A cells; XPG, DNA repair protein complementing xeroderma pigmentosum-G cells; TFIIH, transcription factor II H

More problematic DSBs are primarily repaired by error-free pathway called homologous recombination (HR) operating in the S and G2 phases of the cell cycle and non-homologous end joining (NHEJ), which tends to be error prone and acting in all phases of the cell cycle [90]. Responsibility for genome instability of cells is due to the disruption of many alternative but highly error prone DNA DSB repairs such as single-strand annealing (SSA), microhomology-mediated end joining (MMEJ), and NHEJ [91, 92]. SSA pathway is a mutagenic DSB repair pathway in comparison with error-free HR during S/G2 phases [93]. These error prone DSB repair mechanisms can lead to carcinogenesis [94, 95]. Activation of error prone DNA-repair signalling pathways is involved in the resistance of tumour cells to therapies. An inhibition of these repair mechanisms shows a potential in anticancer treatment [89]. Figure 3 overviews the repair mechanisms, which are involved in the protection against various DNA damage.

## Genoprotective activities of plant natural compounds evaluated in preclinical research

Phytochemicals, either as single substances or their mixtures present in plants, have recently been a highly topical issue of cancer research. Additionally to the significant anticancer properties of plant compounds in already initiated process of carcinogenesis in vitro, in vivo, or in clinical sphere [96–100], their widespread use in cancer chemoprevention should not be forgotten. Plant natural compounds exhibit significant genoprotective effects such as protection against DNA damage, detoxification of carcinogens, or induction of DNA repair [101, 102].

### Antioxidant activity of plant natural substances

#### Non-cancer models

The protective efficacy of whole natural substances against DNA damage was evaluated in several preclinical studies. As examples, we can describe a tropical plant *Chrysobalanus icaco* L. (CHI) exerted antigenotoxicity in peripheral blood cells, antimutagenicity in bone marrow cells and peripheral blood cells, and decreased oxidative stress in peripheral blood neutrophils in Wistar male rats after doxorubicin (DXR)-induced DNA damage and generation of ROS. Chemopreventive efficacy of CHI was noted as an inhibition of NADPH oxidase complex with low levels of DNA damage in rats after DXR exposure. Antunes with co-authors (2016) stated that the genoprotective effect against DXR-induced DNA-damage in vivo was attributed to the phytochemicals and minerals present in CHI exhibiting strong antioxidant properties [103]. Lemongrass essential oil (LEO) that consists mainly of citral (68.78 %) demonstrated several pharmacological, mostly anti-oxidant, and cancer preventive activities. LEO protected human embryonic lung fibroblasts (HELFs) against B[a]P-induced loss of cell viability. Moreover, untreated HELFs exposed to B[a]P showed an increased activity of malondialdehyde (MDA) and a reduced activity of CAT and SOD. Importantly, opposite effect in enzyme activities was observed in HELFs treated with LEO. Additionally, an efficacy of LEO to decrease DNA damage was proven by reduction of 8-OHdG level, indicating LEO as a promising agent in lung cancer chemoprevention [104].

Additionally, *Allium cepa* L. and *A.* × *cornutum* Clementi ex Visiani are common onions which contain large amount of flavonoid quercetin that shows a specific role in different biological processes including cancer [105, 106]. Studies of the DNA protection revealed that the presence of both methanolic extracts of *A. cepa* L. and *A. cornutum* reduced DNA damage, especially DNA strand breaks in human leukocytes. Antioxidant activity was observed in both extracts but slightly higher for *Allium cornutum* due to the higher phenolic content. In DNA nicking assay generated by Fenton reaction, higher concentration of extracts demonstrated significant scavenging activity of free radicals and the ability to maintain DNA in supercoiled structure. Due to high level of quercetin, *Allium cornutum* and *A. cepa* proved to catch and inactivate hydroxyl radicals in H_2_O_2_-induced DNA damage in human leukocytes. On the contrary, higher dose of *Allium cornutum* and *A. cepa* can also provoke oxidative DNA damage mainly by structural characteristics of phenolic compounds which can concurrently play a role as antioxidants and prooxidants. Authors concluded a protective role of *A. cep*a and *A. cornutum* on DNA strand breaks via the antioxidant activity [106]. Furthermore, *Euphorbia dracunculoides* methanol extract (EDME) revealed hepatoprotective and genoprotective effects against oxidative stress and genotoxicity induced with CCl_4_ in the liver of Sprague-Dawley male rats. Administration of EDME in diet of rats increased the levels of antioxidant enzymes (CAT, peroxidase (POD), and SOD), and phase II enzymes (GST and GSH) in liver tissues after CCl_4_ injection compared with control. Similarly, EDME decreased the level of lipid peroxides such as thiobarbituric acid reactant substances (TBARS), nitrite, and hydrogen peroxide in liver tissue. Moreover, decline in the level of DNA damage, including DNA strand breaks caused by CCl_4_, was observed in hepatocytes of EDME-treated rat group compared with control [107].

#### Cancer models

*Brachystegia eurycoma* (BE) from South Eastern Nigeria also known as “Achi” prevented N-methyl-N-nitrosourea (MNU)-induced increasement of MDA and carcinoembryonic antigen (CEA). Chemopreventive study on male Wistar rats demonstrated the hard-mucosal ulceration in the colon associated with inter-glandular inflammation in control group with a single treatment of MNU. On the other hand, only moderate or even no inflammation in the colon in the MNU groups pretreated with BE was detected. Furthermore, studies on the colon revealed that the BE pretreatment to great extent prevented MNU-induced DNA damage to mismatch repair gene MutL homolog1 (MLH1) [108], which mutations are considered biomarkers of carcinogenesis [23]. Furthermore, histological analysis showed oxidative damage in the MNU control group, but not in BE pretreated groups. Moreover, a good correlation was detected between the MLH1 expression and the CEA (*r* = 0.361, *p* < 0.05). Results suggested strong genoprotective activity in the colon via the high antioxidant potential of BE intake [108].

Another study, focused on bioactive compounds of *Butea monosperma* (Lam.) which contains chalcones butein, and isobutrin, and flavonoid butrin, demonstrated anticancer activities [109, 110]. An antioxidant activity of different extracts of *B. monosperma* was measured by DPPH assay. Chloroform and ethyl acetate extracts exhibited pronounced antioxidant activity. Both extracts were shown to protect plasmid DNA of *E. coli* plasmid pBR322 against hydroxyl radical induced DNA damage. Extracts also exerted antiproliferative properties and apoptotic activity through the cell cycle arrest in G1 and sub-G1 phase, enhanced ROS levels, and induced DNA DSBs in MCF-7 breast carcinoma cells [111]. Furthermore, extracts from wines and grapes are rich in polyphenols which can modulate colonocyte mutagenesis and subsequently prevent tumour initiation and promotion [9]. A powdered red wine pomace seasonings (RWPSs) (Sk-S: seedless, W-S: whole, Sd-S: seeds) demonstrated the genoprotective effects against oxidative DNA damage in HT-29 cells [112]. An ability to prevent DNA against oxidative damage via RWPSs was enhanced progressively along digestion resulting in differences from undigested (UD) to colonic fermented (CF) fractions for W-S and between all fractions for Sd-S. In addition to genoprotective effects, the study also determined the antigenotoxic and antiproliferative efficacy of RWPSs in HT-29 colon cancer cells with potential in cancer chemoprevention [112].

Glucosinolates, abundantly presented in *Brassica* vegetables, are hydrolysed during digestion to different products including indole-3-carbinol (I3C). Under acidic condition. I3C polymerizes to indolo[3,2-b]-carbazole (ICZ) [113] with potential anticancer efficacy. An influence of ICZ in the protection against oxidative DNA damage was evaluated in Caco-2 colon carcinoma cell line. The level of DNA strand breaks after pretreatment with ICZ and subsequent exposition to genotoxins t-BOOH, H_2_O_2_ or B[a]P that produce oxidative stress was diminished in Caco-2 cells. On the other hand, simultaneous addition of ICZ did not protect against t-BOOH-induced strand break formation leading to disproof of the direct radical scavenging effect. Interestingly, ICZ did not play a role in SSB repair through the BER, which was not associated with altered expression of BER proteins such as OGG1, APE, PARP1, and XRCC1 in Caco-2 cells. Moreover, ICZ activated ROS scavenging effect with proven low level of 8-OHdG in pretreated Caco-2 cells; however, the antioxidant response pathway of ICZ was independent of Nrf2. It was demonstrated that ICZ activated AhR being protective against oxidative DNA damage in Caco-2 cells. The results suggest that the AhR-dependent pathway was associated with protective activity against ROS-induced DNA damage in colorectal carcinogenesis, and was independent of the Nrf2 pathway [20].

MDA is often used as a marker of the oxidative damage of lipids by free radicals. Recently, we have found that dietary administered clove buds, thyme, and cinnamon, rich in antioxidants, decreased the MDA level in mammary cancer cells in vivo [2, 7, 114–116]. Moreover, young barley leaves and fruit peels polyphenols also lowered the levels of dityrosines, the product of oxidative stress, in mammary cancer cells in vivo [114]. Our results indicated significant antioxidant effects of these plant natural substances in rat carcinoma models and indicated potential genoprotective mechanisms and chemopreventive activity in mammary carcinogenesis.

### Detoxification processes via plant natural substances

#### Non-cancer models

Administrating *Crataegus songarica* methanol extract (CSME) to rats demonstrated antitumour activity, protection against DNA damage, and protection of the kidney and heart tissue against CCl4-induced toxicity. CSME decreased serum creatinine, urea, cholesterol, and MDA level in the kidney and heart tissue. The suppression of GSH, GR, GPx, and GST enzyme activity was also detected in both the kidney and heart tissue. In addition, CSME showed protective effect against CCl_4_-induced DNA damage of kidney and heart tissue [117].

#### Cancer models

Sage tea (*Salvia officinalis*, SO) demonstrated a chemopreventive effect against azoxymethane (AOM)-induced preneoplastic lesions of colon cancer in vivo. Comet assay revealed that SO treatment protected DNA against AOM-induced or H_2_O_2_-induced damage in colonocytes isolated from female rats. Moreover, SO decreased the proliferation marker Ki67 in the colon. Interestingly, effects of SO in the inhibition of cancer initiation were not proven by increased excretion through GST induction or decreased bioactivation by cytochrome P450 2E1 (CYP2E1) in liver homogenates. Results indicated that the consumption of SO tea may prevent the development of colon cancer via various mechanisms such as protection against DNA damage and modulation of cell proliferation resulting in prevention of mutations and their fixation through the cell replication [118]. *Thymus revolutus* Célak essential oil (TRCEO) and its two main constituents, γ-terpinene and p-cymene, have been demonstrated as potential as oxidative agents in lung cancer cells (H1299 and A549) and epidermoid carcinoma cells (A431). Activity of antioxidant enzymes in cancer cells was depended on concentration of TRCEO components and prooxidant/antioxidant effects of extract. IC_50_ and IC_70_ concentrations of TRCEO, γ-terpinene, and p-cymene caused oxidative stress. Parental H1299 cells were the most sensitive to the cytotoxic effects of all tested compounds. The highest membrane and DNA damages were observed in A431 and A549 cells, respectively. TRCEO, γ-terpinene, and p-cymene increased the MDA levels and 8-OHdG formation in all cancer cells used. Additionally, they increased glutathione reductase (GRx), glutathione peroxidase (GPx), GST, and glucose 6-phosphate dehydrogenase (G6PD) activity. These compounds demonstrated antitumour and prooxidative effects with subsequent induction of cytotoxic death of cancer cells [119]. Flaxseed consumption reduced oxidative stress and inflammation in tobacco smoke carcinogen (NNK)-induced lung tumourigenesis in A/J mice. Noteworthy, 10 % flaxseed in the diet altered expression of several CYPs, GSTs, and UDP-glucuronosyltransferase (UGTs). Flaxseed also reduced expression of pro-inflammatory cytokines (IL-1α, IL-6, IL-8, and IL-9) and increased the expression of anti-inflammatory cytokine (IL*-*12α) in lung tissues suggesting chemopreventive properties of flaxseed elicits and associated detoxification of NNK [120].

Methanol extract of *Pongamia pinnata* seeds (MEPPS) and its secondary metabolites pongapin and lanceolatin B showed chemopreventive potential due to their ability to inhibit CYP1A1 and protect CYP1A1-overexpressing HEK293 human embryonic kidney cells against B[a]P-induced toxicity. Additionally, MEPPS and its metabolites also induced the G_0_-G_1_ phase cell cycle arrest of CYP1A1-overexpressing MCF-7 breast cancer cells, and suppressed cyclin D1 levels leading to cellular senescence [121].

### DNA repair mechanisms via plant natural substances

#### Non-cancer models

Several studies were focused on genoprotective activities associated with various plant extracts. Extract of *Sechium edule* (SEE) fruit from *Cucurbitaceae* family is rich in amino acids, saponins, sugars, and flavonoids [122]. Genoprotective activities of SEE against UVA-induced DNA damage in normal human keratinocytes (NHK) were evaluated. SEE increased DNA repair capacities, the maintenance of proliferation, and preservation of stemness property of NHK after UVA exposure. Furthermore, SEE reduced UVA-induced oxidative DNA damage by 30 % compared with control. Oxidised DNA bases modified by ROS in NHK with SEE treatment were repaired via the BER pathway. The upregulation of BER genes OGG1 and MYH in mRNA levels was detected; however, the expression levels of APE1, POL β, LIG3, UNG, and XRCC remained unchanged. Noteworthy, global DNA repair pathways were influenced not only through BER but also through the NER pathway with positive impact on the repair of small oxidative DNA lesion and photoproducts in NHK. Moreover, an amount of CPD, predominant DNA lesions after UVA irradiation in human skin [123], was undoubtedly increased in untreated NHK. On the contrary, number of CPD after UVA exposure was reduced in NHK treated with SEE suggesting the stimulation of repair of such photoproducts [124]. *Ficus carica* leaf extract (FCE), which is native to Egypt or Western Asia, reduced DNA damage and reversed non-steroidal estrogen (DES)-induced DNA damage, especially strand breaks in non-tumourigenic MCF10A human breast epithelial cell line. FCE stimulated DNA repair and optimised comet formation resulting from the irreversible interaction of oxidative quinine metabolites of DES (DESQ) with the nuclear apparatus causing DNA damage [125], keeping in mind DES binds to both estrogen receptors ER-ɑ and ER-β [126]. Overall, the study demonstrated both a chemopreventive and cancer therapeutic role of FCE in early-stage breast cancer [125].

#### Cancer models

*Hemidesmus indicus* (L.), also known as Indian sarsaparilla, is an Indian weed with healing effects of the crude root in Ayurvedic medicine [127]. Study evaluating the cancer chemopreventive and therapeutic potential demonstrated the genoprotective effects of *H. indicus* hydro-alcoholic extract (HIHE) in pre-, co-, and post-treatment in DLD1 colorectal adenocarcinoma cells and in CCRC-CEM T-lymphoblastic cells. Genoprotective properties were evaluated after exposure to genotoxic agents such as etoposide, DXR, and H_2_O_2_. HIHE soxhlet strongly reduced the genotoxicity of etoposide associated with a reduction up to 47.3 %, 42.6 %, and 29.2 % in the H2AX phosphorylation in the pre-, co-, and post-treatment, respectively. Similar effect was observed after treatment with DXR and H_2_O_2_. HIHE pre-treatment played a critical role in genoprotective activity against DNA damage. Additionally, HIHE significantly decreased the cell viability and cell proliferation demonstrated via reduced Ki67 levels in DLD1 and CCRC-CEM cells. Moreover, the cell cycle arrest in S phase for low concentration and G2/M arrest for higher concentration was observed after 24-h HIHE treatment in CCRC-CEM cells. Results suggested that several mechanisms can be involved in genoprotective activity of HIHE by reducing the rates of absorption and uptake of the genotoxic agent and by modulation of DNA repair, cell cycle, or apoptosis at extra- and intracellular level [128].

Isolated phytochemicals from plants also demonstrated genoprotective action in cancer chemoprevention and treatment. FANCA proteins are key players in the canonical Fanconi anemia (FA) repair pathway with impaired response to DNA damage through the HR. Predisposition to breast cancer, including mutation in BRCA1 and BRCA2 (one of the FA protein) gene, is mainly due to disruption of many DNA DSB repairs. High FANCA expression supports the survival of cancer cells despite the DNA damage with subsequent genomic instability via SSA repair. Withaferin A (WA) is a steroidal lactone isolated from winter cherry (*Withania somnifera* (L.) Dunal) that has an ability to reduce FANCA protein levels and downregulates HSP90 expression in MDA-MB-231, SUM-149, MCF-7 breast cancer cells, and U2OS osteosarcoma cells as a platform of DSB repair reporter assays which demonstrated the disrupted interaction between FANCA-HSP90. Additionally, these processes were associated with a defect in WA-induced SSA repair, abolition of FANCD2 monoubiquitination, increased sensitivity to mitomycin C leading to accumulation of DSBs. Despite that FANCA and RAD52 are the major catalytic factors in the SSA subpathway of DNA DSB repair, WA reduced only FANCA but not RAD52 level. In conclusion, defect in SSA repair induced by WA is dependent on the FANCA protein absence. Moreover, overexpression of exogenous WT-FANCA protein complements the repair defect in WA-treated cells [21].

Taken together, the above studies demonstrate that plant natural substances exert potential genoprotective activities in vitro and in vivo, which suggest their application in chemoprevention of various cancer types (Table 2).

**Table 2:** Genoprotective activities of plant natural substances in preclinical non-cancer and cancer models

Genoprotective mechanism	Model of study	Plant natural substances	Study design	Inducers of DNA damage	Genopreventive activities	References
Antioxidant activity	Non-cancer	*Chrysobalanus icaco*	Peripheral blood cells, bone marrow cells, and peripheral blood neutrophils of Wistar male rats	DXR	↓ NADPH oxidase complex, ↓ DNA damage	[103]
		Lemongrass essential oil	Human embryonic lung fibroblasts (HELFs)	B[a]P	↓ MDA, ↑ CAT, ↑ SOD, ↓ 8-OHdG	[104]
		*Allium cepa* and *A.* × *cornutum* methanolic extract	Human leukocytes	H_2_O_2_	↑ Scavenging activity of free radicals, ability to maintain DNA in supercoiled structure	[106]
		*Euphorbia dracunculoides* methanol extract	Liver tissues and hepatocytes of Sprague-Dawley male rats	CCl_4_	↑ CAT, ↑ POD, ↑ SOD, ↑ GST, ↑ GSH, ↓ TBARS, ↓ nitrite and hydrogen peroxide, ↓ DNA damage (DNA strand breaks)	[107]
	Cancer	*Brachystegia eurycoma*	Male Wistar rats (colon cancer model)	NMU	↓ MDA, ↓ CEA, prevention against DNA damage to mismatch repair gene (MLH1)	[108]
		*Butea monosperma* extracts	MCF-7 breast carcinoma cells	Hydroxyl radical	↑ ROS level, ↑ DNA DSBs	[111]
		Powdered red wine pomace seasonings	HT-29 colon cancer cells	Oxidation agent (menadione)	↓ Oxidative DNA breakage, ↓ RONS, indirect antioxidant mechanisms	[112]
		Indolo[3,2-b]-carbazole	Caco-2 colon carcinoma cells	t-BOOH, H_2_O_2_ or B[a]P	↑ Direct radical scavenging effect except for t-BOOH-induced strand breaks, ↓ level of 8-OHdG, activation of AhR-dependent pathway	[20]
		Young barley leaves	Sprague-Dawley female rats (mammary carcinoma model)	NMU	↓ dityrosines	[114]
		*Syzygium aromaticum*	Sprague-Dawley female rats (mammary carcinoma model)	NMU	↓ MDA	[7]
		*Thymus vulgaris*	Sprague-Dawley female rats (mammary carcinoma model)	NMU	↓ MDA	[2]
		Fruit peel polyphenols	Sprague-Dawley female rats (mammary carcinoma model)	NMU	↓ Dityrosines, 3-nitrotyrosine	[115]
		*Cinnamomum zeylanicum*	Sprague-Dawley female rats (mammary carcinoma model)	NMU	↓ MDA	[116]
Detoxification of carcinogens	Non-cancer	*Crataegus songarica* methanol extract	Kidney and heart tissue of male Wistar rats	CCl_4_	↓ MDA, ↓ GSH, ↓ GR, ↓ GPx, ↓ GST, ↓ DNA damage	[117]
	Cancer	*Salvia officinalis*	Isolated colonocytes from female rats (colon cancer model)	AOM and H_2_O_2_	modulation of cell proliferation; not proven by ↑ GST and ↓ bioactivation by CYP2E1	[118]
		TRCEO, γ-terpinene and p-cymene constituents	Lung cancer cells (H1299 and A549) and epidermoid carcinoma cells (A431)	Oxidation agent	↑ Membrane damage in A431 cells ↑ DNA damage in A549 cells, ↑ MDA, ↑ 8-OHdG, ↑ GRx, ↑ GPx, ↑ GST, ↑ G6PD	[119]
		Flaxseed	Lung tumourigenesis of A/J mice	NNK	↓ CYPs, ↑ GSTs, ↑ UGTs	[120]
		Methanol extract of *Pongamia pinnata* seeds, pongapin and lanceolatin B	HEK293 human embryonic kidney cells, MCF-7 breast cancer cells	B[a]P	↓ CYP1A1	[121]
DNA repair	Non-cancer	*Sechium edule* extract	Normal human keratinocytes (NHK)	UVA	↑ Repair capacity (BER, NER), ↓ oxidative DNA damage, ↑ OGG1, ↑ MYH, ↓ CPDs	[124]
		*Ficus carica* leaf extract	Non-tumourigenic MCF10A human breast epithelial cell line	DES	↑ DNA repair	[125]
	Cancer	*Hemidesmus indicus* hydro-alcoholic extract	DLD1 colorectal adenocarcinoma cells and in CCRC-CEM T-lymphoblastic cells	ETP, DXR and H_2_O_2_	↓ Uptake of the genotoxic agent absorption, ↑ antioxidant activity, ↑ modulation of DNA repair, cell cycle, or apoptosis at extra- and intracellular level	[128]
		Withaferin A	MDA-MB-231, SUM-149, MCF-7 breast cancer cells and U2OS osteosarcoma cells	MMC	↓ FANCA protein levels, ↓ SSA error-prone repair, accumulation of DSBs	[21]

*DXR* doxorubicin; *B[a]P* benzo[a]pyrene; *UVA* ultraviolet A; *DES* diethylstilbestrol; *H*_*2*_*O*_*2*_ hydrogen peroxide; *CCl4* carbon tetrachloride; *NMU* N-methyl-N-nitrosourea; *NNK* 4-(methylnitrosamino)-1-(3-pyridyl)-1-butanone; *AOM* azoxymethane, *ETP* etoposide; *MMC* mitomycin C; *t-BOOH* tert-butyl hydroperoxide; *NADPH* nicotinamide adenine dinucleotide phosphate; *MDA* malodialdehyde; *CAT* catalase; *POD* peroxidase; *SOD* superoxide dismutase; *8-OHdG* 8-hydroxydeoxyguanosine; *BER* base excision repair; *NER* nucleotide excision repair; *OGG1* 8-oxoGua DNA glycosylase 1; *MYH* mutY DNA glycosylase; *CPDs* cyclobutane pyrimidine dimers; *CEA* carcinoembryonic antigen; *MLH1* MutL homolog1; *GST* glutathione S-transferase; *GSH* glutathione; *TBARS* thiobarbituric acid reactant substances; *GR* glutathione reductase; *GPx* glutathione peroxidase; *GRx* glutaredoxin; *G6PD* glucose 6-phosphate dehydrogenase; *CYPs* cytochrome P450; *CYP2E1* cytochrome P450 2E1; *CYP1A1* cytochrome P450, family 1, subfamily A, polypeptide 1; *UGTs* UDP-glucuronosyltransferase; *RONS* reactive oxygen/nitrogen species; *DSBs* double-strand breaks; *SSA* single-strand annealing; *AhR* aryl hydrocarbon receptor; *TRCEO Thymus revolutus* Célak essential oil

↑Increase/induce

↓Decrease/inhibit

### Plant bioactive compounds formulated as nanoparticles

Nanotechnologies represent modern multidisciplinary and interdisciplinary approach connected with many applications in scientific research [129, 130]. As detailed above, phytochemicals have a promising potential for human health in preventing or treating various civilisation diseases, including malignant transformation [99, 131, 132]. However, few undesirable aspects, such as their low solubility in water, low stability, or some side effects, are associated with higher doses of plant bioactive compounds and limit their application [133–135]. However, nanoparticles are able to eradicate these processes [129], which are important in the maintenance of genomic stability. Consequently, some studies predicted that green synthesis of nanoparticles is an innovative and future treatment strategy against tumour initiation, promotion, or progression [136–139].

Recently, an antigenotoxic effect of biosynthesised silver nanoparticles (SNPs) of *Ocimum sanctum* leaf extract against cyclophosphamides (oxazaphosphorines acting as the alkylating agents) has been analysed. Biosynthesised nanoparticles of *O. sanctum* exerted a protective effect on the human lymphocytes after cyclophosphamide intervention. SNPs at concentrations of 50, 100, and 200 μl/ml demonstrated a significant decrease in chromosomal damages compared with controls. Higher level of a mitotic index of treated lymphocytes was also observed as a consequence of the protection role green SNPs in genome stability [25]. Genoprotective abilities of green-synthesised selenium nanoparticles of *Terminalia arjuna* leaf extract against arsenic-induced genotoxicity and cell death were analysed in human lymphocytes. Acquired data revealed a reduction of arsenic-induced DNA damage in lymphocytes after the treatment by selenium nanoparticles [22]. Moreover, genoprotective effect of leaf extract of lemon plants used in green-synthesised colloidal selenium nanoparticles was demonstrated after UV-induced DNA damages in human lymphocytes, and could be used as potential chemotherapeutic tool [140]. Also, apigenin combined with poly-(lactide-co-glycolide) acid (PLGA) nanoparticles decreased the chromosomal aberrations in B[a]P- and UVB-induced skin cancer in vivo [19]. Interestingly, the bioavailability of tea polyphenols (TPs) was improved by chitosan and bovine serum albumin (BSA) nanoformulation in mice. Animals were fed by nanoparticles for 3 days before irradiation exposure leading to enhancement of DNA stability via the reduction of oxidative damage [141]. Similarly, silver nanoparticles in combination with glycyrrhizic acid protected Swiss albino mice against ionising radiation, by decrease of DNA strand breaks formation and increase in their repairs [142]. Moreover, theaflavin (TF) and epigallocatechine-3-gallate (EGCG) in PLGA nanoparticles exhibited a higher DNA protection compared with TF and EGCG without nanocarrier in 7,12-dimethybenz[a]anthracene (DMBA)-induced DNA damage in mouse skin [143]. Finally, PLGA-encapsulated *Phytolacca decandra* showed the chemopreventive effect with impact on DNA fragmentation, comet tail length, and level of biomarkers such as NFκB, p53, PARP, ROS generation, CYP1A1, and caspase-3 in mouse model intoxicated with B[a]P + sodium-arsenite [144].

Paradoxically, potential toxicity based on DNA oxidative damage is associated with silver nanoparticles used in many areas, including food, medical, and healthcare [145–147]. However, phytochemicals could counter this effect, as it was described by the use of methanolic and aqueous extract of *Gentiana asclepiadea*, which prevented toxicity of silver nanoparticles treatment in human kidney HEK293 cells [148].

## Clinical research evaluating genoprotective activities of phytochemicals

Consumption of diet rich in substances of plant origin, such as cruciferous vegetable, kiwifruits, fruit juice, or diet of Mediterranean pattern, as well as beverages including tea or coffee, is associated with potent cancer preventive properties. The importance of genoprotective abilities of plant substances was evaluated in clinical, mainly preventive studies conducted on healthy or high-risk individuals [18, 24, 26, 149–158].

### Antioxidant properties of phytochemicals

A mixed berry juice rich in anthocyanin/polyphenolics decreased oxidative DNA damage and increased level of reduced glutathione (GSH) and glutathione status in cells of healthy participants while the observed decrease in oxidative DNA damage may be associated with direct antioxidant effects such as ROS scavenging or chelation [150]. Similarly, the decrease in cancer risk due to the reduction of lymphocyte DNA damage and alterations in the blood antioxidant status in healthy adults was associated with the consumption of watercress-based supplementation. Interestingly, the beneficial effects of the supplementation were more significant in smokers than in non-smokers [149]. Furthermore, kiwifruit protected healthy non-smokers against oxidative DNA damage, upregulated the level of antioxidant activity, and stimulated DNA repair, which appears to be a result of an increased stability of the OGG1 protein or availability of an unknown co-factor and not by altered gene expression of OGG1 or APE1. Interestingly, these changes were not related to the number of kiwifruits consumed [18]. Three-month administration of the antioxidants ascorbic acid (vitamin C) and D-α-tocopherol (vitamin E) in volunteers with history of melanoma or no UV-induced skin cancer increased antioxidant capacity of skin as demonstrated by a significant decrease in thymine dimmers induced by the UVB irradiation [158]. Additionally, green tea polyphenols (GTP) decreased level of 8-OHdG in individuals with high risk of liver cancer [151].

### Phytochemicals protecting against carcinogen-induced DNA damage

Cooked *Brassica carinata* (no allyl isothiocyanate, AITC) reduced DNA damage induced by aflatoxin B1 (AFB1) in peripheral blood mononuclear cells of healthy participants exposed to aflatoxin B1 with or without metabolic activation using human S9 mix when compared with baseline. Raw *B. carinata* (AITS-containing) led to the reduction of DNA damage by S9 activated AFB1 only. However, any changes in plasma antioxidant capacity were not observed in any group, which was possibly a result of no changes in the content of total polyphenols due to cooking procedure. Moreover, the bioavailability of phenolics may increase as a result of short-term cooking of some vegetables. Above all, antigenotoxic efficacy of *B. carinata* in healthy participants is not primarily mediated by AITS due to the absence of AITC metabolites in plasma or urine of subjects who consumed the cooked *B. carinata* leaves [152]. Further, the consumption of a hop flavonoid xanthohumol (XAN) was associated with preventive properties against DNA damage induced by dietary carcinogens in healthy non-smokers. XAN substantially decreased B[a]P- and 2-amino-3-methylimidazo[4,5-f]quinoline (IQ)-induced DNA damage while moderate protective effects were associated with N-nitrosodimethylamine (NDMA) suggesting an important protective efficacy of XAN against carcinogens detoxified by α-GST that represents major groups of dietary carcinogens [154]. Moreover, coffee induced GSTP, a member of GST isoenzymes, and protected lymphocytes of healthy subjects against DNA damage induced by (+/-)-anti-B[a]P-7,8-dihydrodiol-9,10-epoxide (BPDE), the DNA-reactive metabolite of B[a]P [155]. Similarly, brussel sprouts protected peripheral human lymphocytes of healthy participants against 2-amino-1-methyl-6-phenylimidazo[4,5-b]pyridine (PhIP) possibly via the inhibition of sulfotransferase 1A1, a key enzyme participating in the PhIP activation. Moreover, the reduced oxidative DNA damage due to the presence of compounds acting as direct ROS scavengers was also attributed to the consumption of sprouts [156].

High-phenol extra-virgin olive oil (EVOO) could be at least partially associated with lower mortality or incidence of cancer in regions characterised by Mediterranean diet. Actually, the reduction of DNA damage was observed in healthy postmenopausal women after the consumption of a high-phenol EVOO [24]. Similar results were observed after a consumption of carotenoid supplementation suggesting that the combination of carotenoids (in doses easily achieved by the diet) possesses protective abilities against DNA damage [26]. Anticarcinogenic properties of cruciferous vegetables are associated with improvement in cell protection against DNA damage. The study evaluating protective effects of broccoli in healthy young male revealed significant decrease in strand breaks in both smokers or nonsmokers and reduction of oxidised purines in smokers [157].

Accordingly, an intake of natural substances, which exert potential genoprotective properties and subsequent chemopreventive abilities against several cancer types, should represent a significant and important element of everyday diet. Due to the presence of health-threatening compounds in the human environment, we emphasize a significant protective effects of phytochemicals against damage leading to carcinogenesis in both healthy and high-risk individuals (Table 3).

**Table 3:** Genoprotective properties of plant natural substances in clinical cancer research

Genoprotective mechanism	Natural substance/dietary supplement	Study design	Participants characteristic (number of participants)	Group distribution, dosage	Genoprotective activities	Reference
Antioxidant activity	Anthocynin/polyphenolic-rich fruit juice		Healthy non-smoking men (*n* = 27)	Fruit juice (*n* = 18, 700 ml/daily); Control (*n* = 9, polyphenol depleted juice) for 4 weeks	Fruit juice: ↓ oxidative DNA damage ↑ GSH ↑ GSH status	[150]
	Watercress	A single-blind, randomised, crossover study	Healthy smokers (*n* = 30), healthy non-smokers (*n* = 30)	Watercress supplementation: 85 g/daily or control for 8 weeks	Watercress supplementation: ↓ basal DNA damage (17 %; *p* = 0.03), ↓ basal plus oxidative purine DNA damage (23.9 %; *p* = 0.002) ↑ plasma lutein (by 100 %) ↑ plasma β-carotene (33 %) (*p* < 0.001)	[149]
	Kiwifruits	A randomised crossover study	Healthy non-smokers (*n* = 14)	1, 2, or 3 kiwifruits/daily each volunteer	↑ Antioxidant status of plasma and lymphocytes (↓ DNA breaks) ↓ levels of endogenous oxidation of pyrimidines and purines in DNA ↑ DNA repair activity	[18]
	Ascorbic acid (vitamin C) and D-α-tocopherol (vitamin E)		History of melanoma, BCC or SSC (*n* = 14), no UV-induced skin cancer (*n* = 4)	Ascorbic acid 2 g/daily and D-α-tocopherol 1000 IU/daily for 90 days	↓ Sunburn reaction to UVB irradiation ↓ thymine dimers	[158]
	GTP	Phase IIa randomised, double-blinded and placebo-controlled chemopreventive trial	High-risk individuals of liver cancer (*n* = 124)	Low dose (500 mg GTP/daily, *n* = 42), high dose (1000 mg GTP/daily, *n* = 41), or placebo (*n* = 41)	↓ DNA damage (↓ 8-OHdG level)	[151]
Protection against exposure to carcinogens	Ethiopian kale (*Brassica carinata*)	Randomised, single blind, controlled crossover intervention trial	Healthy participants (*n* = 22)	Cooked *B. carinata* group (*n* = 11); Raw *B. carinata* group (*n* = 11) for 5 days (15 g of freeze-dried *B. carinata* leaves/daily cooked/unprocessed)	Cooked *B. carinata*: ↓ AFB1-induced DNA damage (+S9 mix: 35%, −S9 mix: 33 %, *p* ≤ 0.01) Raw *B. carinata*: ↓ AFB1-induced DNA damage (+S9: 21 %, *p* = 0.08)	[152]
	XAN	Crossover placebo-controlled trial	Healthy non-smokers (*n* = 22)	XAN-beverage (12 mg of xanthohumol, *n* = 11); placebo (*n* = 11) for 2 weeks (1l/daily)	XAN: → α-GST	[154]
	Coffee		First trial (*n* = 10)	Unfiltered coffee (1l/daily over 5 days)	→ GSTP	[155]
			Second trial (*n* = 14)	Unfiltered and paper filtered coffee (1l/daily over 3 days)	→ GSTP	
			Third trial (*n* = 7)	Unfiltered coffee (1l/daily over 5 days)	↓ BPDE-induced DNA migration	
	Brussel sprouts	Intervention study	Healthy participants (*n* = 8)	Brussel sprouts for 6 days (300 g/daily)	Brussel sprouts: ↓ PhIP-induced DNA migration (97%) ↓ endogenous formation of oxidised bases ↓ hydrogen peroxide-induced DNA damage (39 %)	[156]
DNA damage protection	High-phenol EVOO	Randomised crossover intervention trial	Healthy postmenopausal women (*n* = 10)	High-phenol EVOO (592 mg total phenols/kg), low-phenol EVOO (147 mg/kg) 50 g/daily	↓ DNA damage by 30% with high-EVOO vs. low-EVOO	[24]
	Carotenoid supplementation	Randomised, double-blind, placebo-controlled intervention study	Healthy postmenopausal non-smoking women (*n* = 37)	Daily dose of mixed carotenoids (β-carotene, lutein, and lycopene; 4 mg each), 12 mg of a single carotenoid (beta-carotene, lutein, or lycopene), or placebo for 56 days	All carotenoid supplemented groups: ↓ endogenous DNA damage	[26]
	Broccoli	Randomised crossover study	Healthy young smokers and non-smokers (*n* = 20)	Broccoli (200 g/daily) or controlled diet for 10 days	↓ Strand breaks in smokers and non-smokers (− 22.2%; *p* < 0.0001) ↓ oxidised purines in smokers (− 51.0%; *p* < 0.0001)	[157]

*AFB1* aflatoxin B1; *BCC* basal cell carcinoma; *BPDE* (±)-anti-B[a]P-7,8-dihydrodiol-9,10- epoxide; *EVOO* extra-virgin olive oil; *GSH* reduced glutathione; *GSTP* glutathione S-transferase P; *GTP* green tea polyphenols; *HCA* heterocyclic amines; *SSC* squamous cell carcinoma; *UV* ultraviolet radiation; *UVB* ultraviolet B; *XAN* xanthohumol; *α-GST* glutathione S-transferase α; *8-OHdG* 8-hydroxydeoxyguanosine

↑increase

↓decrease

→induction

**Fig. 4:**
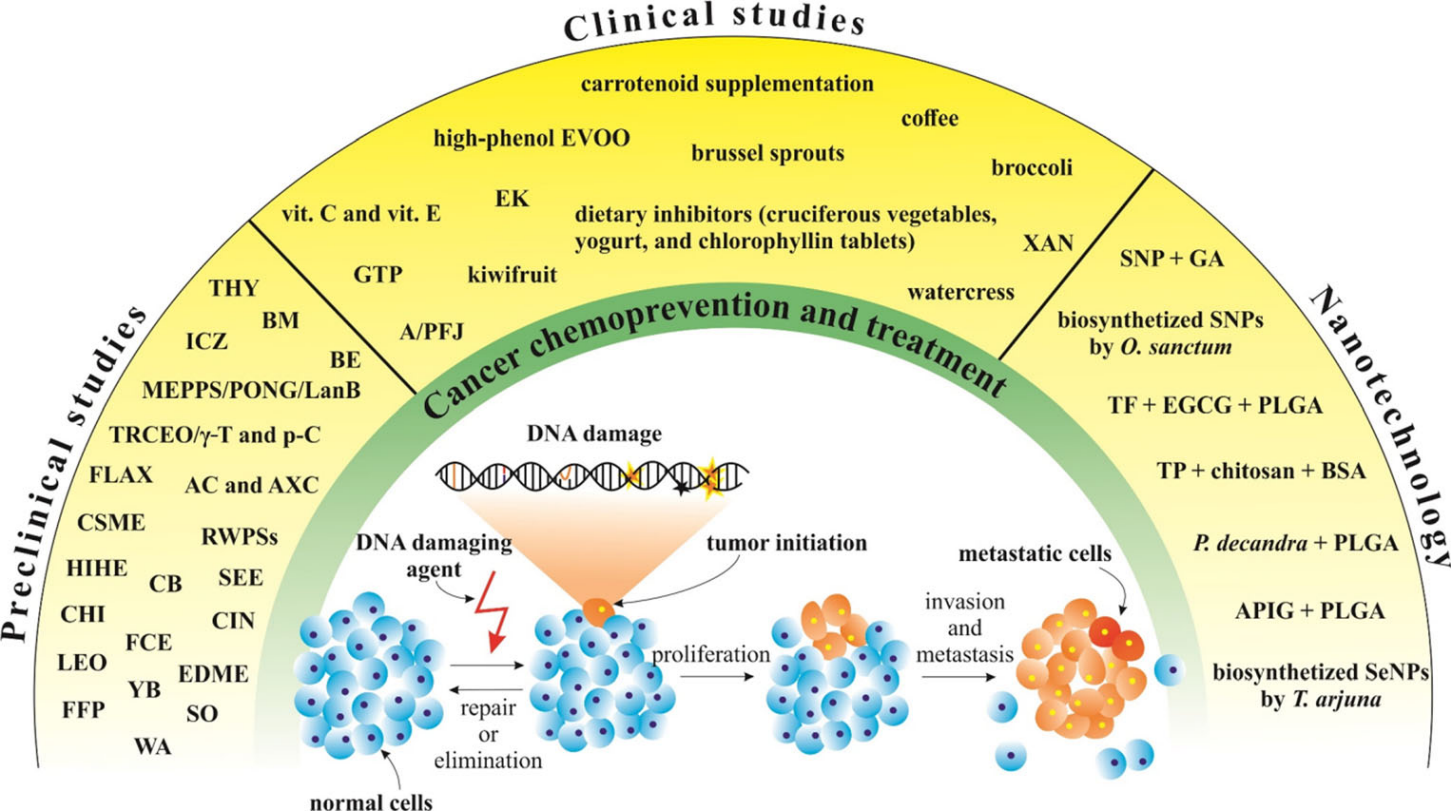
Overview of plant natural substances which are associated with genoprotective abilities in preventive and therapy studies. SO, *Salvia officinalis*; RWPSs, powdered red wine pomace seasonings; LEO, lemongrass essential oil; CHI, *Chrysobalanus icaco*; SEE, *Sechium edule* extract; HIHE, *Hemidesmus indicus* hydro-alcoholic extract; FCE, *Ficus carica* leaf extract; BE, *Brachystegia eurycoma*; AC and AXC, *Allium cepa* L. and *Allium* × *cornutum*; BM, *Butea monosperma*; WA, withaferin A; ICZ, indolo[3,2-b]-carbazole; EDME, *Euphorbia dracunculoides* methanol extract; CSME, *Crataegus songarica* methanol extract; TRCEO/γ-T/p-C, *Thymus revolutus* Célak essential oil/γ-terpinene/p-cymene; FLAX, flaxseed; MEPPS/PONG/LanB, methanol extract of *Pongamia pinnata* seeds/pongapin/lanceolatin B; YB, young barley; CB, clove buds; THY, thyme; FPP, fruit peel polyphenols; CIN, cinnamon; vit. C and vit. E, vitamin C (ascorbic acid) and vitamin E (D-α-tocopherol); GTP, green tea polyphenols; A/PFJ, anthocynin/polyphenolic-rich fruit juice; EK, Ethiopian kale (*Brassica carinata*); EVOO, extra-virgin olive oil; XAN, xanthohumol; SNPs, silver nanoparticles; GA, glycyrrhizic acid; TF, theaflavin; EGCG, epigallocatechine-3-gallate; PLGA, poly (lactide-co-glycolide) acid; TP, tea polyphenols; BSA, bovine serum albumin; APIG, apigenin; SeNPs, selenium nanoparticles

The significant genoprotective abilities of plant natural substances in preclinical, clinical, and nanotechnology approach described above are summarised in Fig. 4.

### Protective and toxic interactions in the clinical setting

The secondary effects of oncological treatments, in terms of chemo- and radiotherapy, and its management have been widely addressed in multiple publications since their very beginnings [159–161]. Different pharmaceutical options, in a variety of presentations, are currently available for toxicity management according to the affected region or tissue, and their use has been included and recommended in various practice guidelines [162, 163]. However, not many studies have addressed the potential benefits of natural compound utilisation as a scientifically validated approach [164]. This resembles an important topic to be evaluated, as depending on factors such as the geographical provenance or cultural background, the association between occidental and traditional medicine could be a factor to consider in treatment decision-making [165, 166]. It should be as well taken into account that nearly 2 % of cancer deaths might be directly related to X-ray exposure; therefore, major efforts to generate further knowledge in this topic are warranted [167].

Several mechanisms to explain the chemo- or radio-protective effect of plant-based management have been postulated. The high polyphenol content found in plants might be one of the substances mediating the cytoprotective effect. Through it, antioxidant enzymes may upregulate mRNA, diminishing the oxidative response after radiation exposure; additionally, DNA repair genes upregulation could contribute ameliorating failures in double-strand repairing [168]. Additionally, generating reactive oxygen species and apoptotic pathways induction has been as well described as associated mechanisms [169].

### Implicancies in radiation protection

On this molecular basis, different studies have been conducted in order to prove its clinical impact. An investigation developed in the setting of cancer staging scans analysed the impact of multi-agent antioxidant pills administrated before Tc99 isotope application, containing ascorbate, n-acetylcystein (NAC), lipoic acid, and beta carotene. DSBs were measured both before and after imaging and compared between a control group and an interventional group. A significant DSB 60 % reduction was observed in the interventional group [170].

The expected profile of radiotherapy toxicity is quite known according to the irradiated tissue, delivered dose, volume of treatment, amongst other factors [171]. Radioprotectors, such as Amifostine, have proven to be efficient in management, for example, of head and neck cancer treatment-related toxicity; however, its availability and implementation in clinical routine due to logistics or elevated costs could impact its accessibility [172, 173]. On the contrary, natural compounds or phytochemicals could be found widely across the globe, although safety, dosing, and outcome data persist to be currently scarce. Preliminary evidence of the effect of coffee (*n* = 75), *Matricaria recutita* (*n* = 52), *Aloe vera* (*n* = 61), and *Calendula officinalis* (*n* = 40) have shown a positive and statistical significant impact in oral mucositis reduction in prospective studies, although it is worth remarking that these investigations might present selection bias [174–176]. In the setting of breast cancer, the protective effect of 6 g per os of curcumine was assessed throughout the course of radiotherapy, randomised in a small cohort of 30 patients. The evaluated endpoint was moist desquamation, which was positive in 28.6 % against 87.5 % for the interventional and control groups, respectively [177].

Negative interactions between plants or phytochemicals and radiotherapy have not been widely addressed and current information regarding this topic remains sparse. In general terms, only low-quality data showing no toxic crossed-interactions has been to date reported [178, 179]. However, caution must be taken for smoker patients during radiotherapy. The administration of antioxidants to this particular subgroups of patients has shown in a randomised trial, that it could carry an increased recurrence and mortality rate risk (HR 2.42 and 2.26, respectively) compared with the control non-interventional group, probably due to an elevation of seric carboxyhemoglobine, thus decreasing the oxygen-dependant radiotherapy effect [180, 181].

In addition, further research is being currently carried towards clarifying the radiosensitizer role of phytochemicals such as flavonoids, in order to enhance radiotherapy´s therapeutical ratio [182].

### Implicancies in chemotherapy

In regard to chemotherapy or combined radiochemotherapy, toxicity rates still represent a recurrent issue to be solved, causing treatment interruptions in detriment of the expected oncological outcomes. A Cochrane systematic review analysed the results of 4 different studies who compared chemotherapy alone against chemotherapy plus traditional Chinese herbal medicine as cytoprotector. With 342 included subjects in total, and although a low-quality level was observed amongst the trials and no robust evidence could be obtained, the collected data points towards an improvement in immunocompetent cell stimulation and side effect decrease. Besides this, no toxic effect was associated to plant usage [183]. This hypothesis-generating conclusion should be considered baseline for further research with adequate methodology.

The willingness of natural medicine utilisation in certain regions ought to be of the oncology practitioners’ knowledge [184, 185]. Due to the wide spectrum and availability of natural products and lack of knowledge dissemination in terms of potential adverse events, caution should be taken while prescribing or delivering treatment. This is supported by an analysis comparing drug- (DDI) and herb-drug (HDI) interactions in cancer care. In these 149 patient cohorts, 36 clinically relevant DDI in 26 patients (17.4 %) and 122 HDI in 84 patients (56.4 %) were detected [186]. The importance of acknowledging the patient’s treatment desires lies in predicting and preventing potential harmful interactions combined with the indicated treatment. Besides, an important number of patients (~ 58 %) tend to hide this information from their treating physicians, which may also increase the risk of developing adverse events [187].

Different mechanisms have been described for explaining potential undesired interactions. The interference in P450 isozyme metabolism or drug transporter P-glycoprotein function due to consumption of garlic (*Allium sativum)*, *Ginko biloba*, *Echinacea purpurea*, ginseng (*Panax ginseng)*, amongst others, has been already mentioned in the literature as potential triggers for hepatic toxicity and reduced anticancer therapy effects [164, 188].

In contrast and reported by two randomised studies, higher seric concentrations of selenium and vitamin E have shown to improve hemathologic toxicity and neuropathy, respectively, in the setting of cisplating administration [189, 190].

### Therapeutic and preventive ratio enhancement

Evidence from different publications, including one systematic review of 49 human studies, have highlighted the non-interfering profile of natural compounds with radiotherapy, such as beta-carotene, vitamins A, B, E, selenium, amongst others. Moreover, a lower rate of secondary effects enhanced tumouricidal action, and even potentially survival benefit [178, 182, 191].

Although widely studied in pre-clinical trials, no strong evidence in human subjects has been collected to date, in to classify phytochemicals usage and dosing in a defined clinical scenario. The example of breast cancer patients has shown, mostly in vitro, different action levels regarding proliferation, apoptosis, and metastasis, for specific compounds. For instance, resveratrol has shown reducing negative features by acting on estrogen receptors (ER), EFGR/PI3K, and ERK1/2 pathways. Additionally, the activity of other phytochemicals, like lignans or curcumin, inhibiting the HER-2 pathway, might be as well of benefit for specific case scenarios [192]. Clinical evidence extracted from other compound usage experience (isoflavones) opens a new path for further research. As it has been reported, a seemly lower rate of breast cancer has been related to its consumption in some Asian countries [193]. In addition, a prospective Spanish trial demonstrated the protective effect of the Mediterranean diet against breast cancer development [194, 195]. Caution while interpreting these publications is suggested, as they might incur in selection bias.

New approaches will be developed in the upcoming years with further vegetal species under the scope. Initial reports of antitumoural activity of *Banisteriopsis caapi*, for example, through various hypotetical molecular pathways, such as N,N-dimethyltryptamine in the intracellular sigma-1 receptors, and the activity of harmine, tetrahydroharmine, and harmaline, are generating great expectations to delucidate their potential contribution in cancer care [196]. Other studies currently undergoing in various institutions, to demonstrate the value of different phytochemicals for specific sites, are eagerly awaited to increase the oncologists’ therapeutical arsenal.

## Chemoprevention exemplified for individual stages of cancer’s risk, development, and progression

As it was detailed above, several plant natural substances demonstrate genoprotective effects by complex antioxidant and detoxification activities, neutralisation of carcinogens, and DNA repair promotion. On the other hand, cytotoxic and genotoxic effects of plants and extracts have been demonstrated towards quickly proliferating tumour cells discriminating them against physiologic tissue [197, 198]. Below paragraphs illustrate clinically relevant effects applicable to

- general cancer prevention
- pre-cancerous lesions—individualised cancer prevention
- selective cytotoxic effects against malignancies
- circulating tumour cells and metastatic disease—mitigating strategies
- phytochemical supplement in optimal palliative care setting

as exemplified specifically for colon carcinomas.

### General colon cancer prevention in the population

Multi-factorial stress conditions leading to the excessive production of ROS are well-acknowledged to cause cancer predisposition as reported for hepatocellular carcinoma, glioma, malignancies of the blood, breast, colorectum, esophagus, kidney, lung, mouth, ovary, pancreas, prostate, and stomach [199]. To this end, the risk of developing malignancies is associated with the dose as well as timing of the exposure [200]. Consequently, individualised predictive approach is essential to make the primary prevention targeted and cost-effective [201, 202].

One of the most ubiquitous stress factors is an imbalanced and/or unhealthy diet increasing the general predisposition to colon cancer [203]. Specifically, so-called Western Diet Pattern is characterised by high intake of processed meat, red meat, or high-fat dairy products [204] being associated with an increased risk of colon cancer [167, 168]. In contrast, the Mediterranean diet pattern based on fruits and vegetables rich in various phytochemicals shows chemopreventive anticancer effects mediated via complex antioxidant, detoxification, and free radical scavenging activities [205, 206]. Unfortunately, people at high risk, due to genetic (inborn family) predisposition to colon cancer are not sufficiently responsing to a dietary prevention [207]. Consequently, supplementary protective measures are currently under extensive consideration such as improved microbiome setup as well as probiotics and nanoparticles with prebiotic properties supportive for immune system and applied depending on the individual predisposition and personalised patient profile [208, 209].

### Pre-cancerous lesions and malignant cell transformation

Pre-cancerous colon lesions, such as polyps, ulcers, erosions, vascular lesions, mass, and nodules, are considered prestages of the colon cancer development [210]. Early diagnosis of multiple adenoma, a precursor to colorectal cancer, followed by application of plant natural substances may be highly protective reducing the overall risk of cancer development [174]. For example, Lynch syndrome patients, who are predisposed to various cancers including colorectal carcinomas and suffering from insufficient DNA repair capacity, demonstrate reduced inflammation and overall cancer risks by intake of nutritional Nrf2 activators such as phytochemicals (sulforaphane, curcumin, quercetin, resveratrol, and EGCG) [211]. Further, progression of neoplastic lesions into colon cancer is suppressed by *Salvia officinalis* tea via its chemopreventive effects [118]. To this end, the transformation of neoplastic lesions into malignant cells and anticancer effects by phytochemicals might be highly selective by killing rapidly proliferating cells [212]. Specifically, *Premna odorata* leaves, *P. odorata* bark hexane fractions and *Artocarpus camansi* leaves were found to be highly cytotoxic against HCT116 human colon cancer cell line, with *P. odorata* bark hexane extract demonstrating high selectivity/cytotoxic index [213]. Similar effects have been demonstrated also for several cancer types: triterpenoids, oleanolic acid 3-acetate, and betulinic acid, isolated from dichloromethane extracts of *Clerodendrum indicum* and *Clerodendrum villosum*, revealed moderate to strong cytotoxicity to colorectal adenocarcinoma (SW620), bronchogenic carcinoma (ChaGo-K-1), hepatocellular carcinoma (HepG2), gastric carcinoma (KATO-III), and ductal carcinoma (BT-474) cancer cell lines [214].

### Cytotoxic effects to suppress circulating tumour cells and metastatic disease

Some plants via their content compounds exhibit selective cytotoxic effects against circulating tumour cells and metastatic potential. Specifically, apigenin, which can be detected in many vegetables and herbal spices, is capable to inhibit epithelial-mesenchymal transition (EMT) of human colon cancer cells that plays an essential role in the cancer metastasis progression [215]. Similarly, natural polyphenol calebin A, a component of turmeric, evidently suppresses proliferation, invasion, and metastasis spread by human colorectal cancer cells [216]. Considering corresponding molecular mechanisms, phytochemicals are capable to perturb signalling pathways associated with the cancer progression. Further clinical studies are needed to understand in more detail how patients can be best stratified for anticancer and anti-metastatic medication by well selected plants and phytochemicals.

### Plant and phytochemical supplement in optimal palliative care setting

The concept of palliative care provided to cancer patients is currently evolving from just end of life care and relieve suffering to include all aspects of cancer survivorship. The new challenging concept needs to thoroughly consider diet and nutrition which positively impact individual outcomes by affecting cancer recurrence and progression. To this end, clear evidence is provided that herbs and their constituent phytochemicals may be biologic response modifiers that could increase cancer control within palliative care [217]. Moreover, herb and dietary supplements are the most popular complementary and alternative medicine modality used by cancer patients. How much they are supportive and whether they may interfere with the efficacy and safety of conventional medicines remains to be further investigated [218].

## Concluding remarks, expert recommendations and outlook

Natural substances represent an attractive strategy for cancer primary and secondary prevention [219–221]. Preclinical cancer research demonstrated potent genoprotective properties of natural plant substances in non-cancer models [103, 106, 124–126, 222]. Regarding clinical research, the uses of germ-line, familiar, or high-risk cohorts are associated with more power over a shorter time frame of the clinical trials when compared with subjects with average risk [223]. Identification of high-risk individuals who would benefit from targeted preventive strategies described above is an essential measure which may significantly reduce cancer incidence in the population. Multi-level diagnostics, including phenotyping and genotyping, is considered an optimal tool for prediction and targeted prevention [224, 225]. Further, multiomics plays a key role in cancer predictive and early diagnostics [226].

By evidence, administration of plant natural substances and/or diet supplements slows down or even inhibits carcinogenesis in healthy and high-risk individuals. Plant natural substances and phytochemicals decrease oxidative damage to biologically important molecules, increase antioxidant status and scavenging capacity against excessive ROS production; thereby, cells are better protected against carcinogen-induced DNA damage and both—detoxification and DNA repair pathways—are stimulated [18, 24, 26, 149–158]. However, clinically relevant recommendations in this regards are still underrepresented. Obviously clinical trials focused on individualised patient profiles and consequent patient stratification would be useful to bring the preclinical discoveries in the field to the daily clinical practice benefiting the patients and healthcare as the whole. To this end, since a large part of cancers is considered preventable, particularly primary chemoprevention may offer plausible solutions to address the global problem of increasing cancer incidence [227, 228]. In general, innovative approaches by preventive medicine provide new opportunities for clinical oncology [193]. 3PM strategies are considered being particularly important to advance the overall cancer management making the services cost-effective [194]. As detailed above, chemopreventive strategies play an important role in the context of 3P medicine [229]. However, further research for determining active compounds and dosing selection is urgently warranted to effectively promote their clinical implementation in terms of primary and secondary chemoprevention as well as cancer management as a whole [230–232].
